# Development of the VEGANScreener, a Tool for a Quick Diet Quality Assessment among Vegans in Europe

**DOI:** 10.3390/nu16091344

**Published:** 2024-04-29

**Authors:** Selma Kronsteiner-Gicevic, Leonie H. Bogl, Maria Wakolbinger, Sandra Müller, Joelina Dietrich, Willem De Keyzer, Vanessa Bullón-Vela, Eliska Selinger, Vanessa Keller, Ainara Martínez Tabar, Tooba Asif, Leone Craig, Janet Kyle, Sabrina Schlesinger, Christian Köder, Anna Ouradova, Marina Henikova, Wendy Van Lippevelde, Monika Cahova, Miguel Angel Martínez González, Walter Willett, Maira Bes-Rastrollo, Jan Gojda, Stefaan De Henauw, Markus Keller, Marek Kuzma, Eva Schernhammer

**Affiliations:** 1 Department of Epidemiology, Center for Public Health, Medical University of Vienna, 1090 Vienna, Austria; 2 Department of Nutritional Sciences, Faculty of Life Sciences, University of Vienna, 1090 Vienna, Austria; 3 Department Nutrition and Dietetics, Faculty of Health Professions, Bern University of Applied Sciences, 3008 Bern, Switzerland; 4 Department of Social and Preventive Medicine, Center for Public Health, Medical University of Vienna, 1090 Vienna, Austria; 5 Research Institute for Plant-Based Nutrition, 35444 Biebertal, Germany; 6 School of Bioscience and Industrial Technology, HOGENT University of Applied Sciences and Arts, 9000 Ghent, Belgium; 7 Department of Public Health and Primary Care, Faculty of Medicine and Health Sciences, Ghent University, 9000 Ghent, Belgium; 8 Department of Preventive Medicine and Public Health, University of Navarra-IdiSNA, 31008 Pamplona, Spainmbes@unav.es (M.B.-R.); 9 Department of Epidemiology and Biostatistics, Third Faculty of Medicine, Charles University, 100 00 Prague, Czech Republic; 10 Center for Public Health Promotion, National Institute of Public Health, 100 00 Prague, Czech Republic; 11 Institute of Applied Health Sciences, University of Aberdeen, Aberdeen AB24 3FX, UK; 12 Institute for Biometrics and Epidemiology, German Diabetes Center, Leibniz Institute for Diabetes Research at Heinrich, Heine University Düsseldorf, 40225 Düsseldorf, Germany; 13 Department of Internal Medicine, University Hospital Kralovske Vinohrady, Third Faculty of Medicine, Charles University, 100 00 Prague, Czech Republic; 14 Faculty of Economics and Business, Department of Marketing Innovation & Organisation Unit Consumer Behaviour, Ghent University, 9000 Ghent, Belgium; 15 Institute for Clinical and Experimental Medicine, 140 21 Prague, Czech Republic; 16 Department of Nutrition, Harvard TH Chan School of Public Health, Boston, MA 02115, USA; 17 Biomedical Research Networking Center for Physiopathology of Obesity and Nutrition (CIBERobn), 28029 Madrid, Spain; 18 Department of Epidemiology, Harvard TH Chan School of Public Health, Boston, MA 02115, USA; 19 Institute of Microbiology of the CAS, 142 20 Prague, Czech Republic; 20 Department of Analytical Chemistry, Faculty of Science, Palacky University, 779 00 Olomouc, Czech Republic

**Keywords:** diet screener, vegan diet, diet assessment, diet quality, screener development, Delphi method

## Abstract

Background: Plant-based diets are not inherently healthy. Similar to omnivorous diets, they may contain excessive amounts of sugar, sodium, and saturated fats, or lack diversity. Moreover, vegans might be at risk of inadequate intake of certain vitamins and minerals commonly found in foods that they avoid. We developed the VEGANScreener, a tool designed to assess the diet quality of vegans in Europe. Methods: Our approach combined best practices in developing diet quality metrics with scale development approaches and involved the following: (a) narrative literature synthesis, (b) evidence evaluation by an international panel of experts, and (c) translation of evidence into a diet screener. We employed a modified Delphi technique to gather opinions from an international expert panel. Results: Twenty-five experts in the fields of nutrition, epidemiology, preventive medicine, and diet assessment participated in the first round, and nineteen participated in the subsequent round. Initially, these experts provided feedback on a pool of 38 proposed items from the literature review. Consequently, 35 revised items, with 17 having multiple versions, were suggested for further consideration. In the second round, 29 items were retained, and any residual issues were addressed in the final consensus meeting. The ultimate screener draft encompassed 29 questions, with 17 focusing on foods and nutrients to promote, and 12 addressing foods and nutrients to limit. The screener contained 24 food-based and 5 nutrient-based questions. Conclusions: We elucidated the development process of the VEGANScreener, a novel diet quality screener for vegans. Future endeavors involve contrasting the VEGANScreener against benchmark diet assessment methodologies and nutritional biomarkers and testing its acceptance. Once validated, this instrument holds potential for deployment as a self-assessment application for vegans and as a preliminary dietary screening and counseling tool in healthcare settings.

## 1. Introduction

Veganism [1,2], which encompasses a philosophy of abstaining from the use of foods, beverages, and non-dietary products derived from animals, has seen a rising trend in Europe, particularly among younger and highly educated populations [3,4]. From a dietary perspective, vegans avoid consuming meat, fish, eggs, dairy products, animal fats (e.g., beef tallow and pork lard), and other substances of animal origin [5]. The shift towards veganism is driven by a myriad of motivations, including concerns for animal welfare, social justice issues, climate change implications, individual health, and personal food and taste preferences [6,7]. This shift is mirrored by the rapid expansion of vegan food markets in Europe, indicating a growing demand for vegan foods [8]. A recent Euromonitor’s Product Claims survey [9] showed a steep rise in vegan claims on processed foods, including processed cheese, meat substitutes, pastries, pizza, ice cream, lollipops, gums, jellies, and sauces in 2020–2021. While official surveillance data on the number of vegans across Europe are lacking, available evidence suggests prevalence rates ranging from 0.1% in Spain [10] and 1% in the Czech Republic [11] to 1.4% in Austria, 2–3% in Belgium [12], and 1.3–4% in Germany [13]. In the 2021 Euromonitor’s Lifestyle Survey [14], 3.4% Europeans stated adherence to a vegan diet. This trend is expected to continue growing due to the increasing public awareness of animal food production processes [7], food-related climate change [15,16], and the inclusion of plant-based and sustainable diets in national dietary guidelines [17]. Vegan diets are associated with a higher intake of fruits, vegetables, nuts, legumes and seeds, dietary fiber, phytochemicals, and a range of vitamins and minerals, and have a lower glycemic load compared to omnivorous diets [18,19]. They are also associated with a range of positive health outcomes, including lower risks of all-cause mortality and coronary heart disease; a reduction in low-density lipoprotein (LDL) cholesterol and apolipoprotein B (apoB) levels; and improved glycemic control, beneficial gut microbiota shift, and greater weight loss [20,21,22,23,24,25]. A recent study found a higher submaximal endurance and oxygen consumption during exercise among vegans [26]. However, vegan diets may be deficient in some key nutrients, primarily vitamins, including riboflavin, niacin, B12, and D; minerals such as iodine, zinc, calcium, iron, and selenium; polyunsaturated fatty acids docosahexaenoic acid (DHA) and eicosapentaenoic acid (EPA); and the amino acid lysine [18,27,28,29]. In addition, vegans may be at a higher risk of bone fracture [20], potentially due to inadequate calcium and vitamin D, of iodine overconsumption in case of frequent consumption of seaweed/kelp [30], and of hemorrhagic stroke, potentially due to a very low intake of saturated fats [31]. While research on the health effects of “healthy” vs. “less healthy/unhealthy” vegan diet patterns is still sparse, studies identified several types of specific diet patterns among vegans [32,33]. Consumption of ultra-processed foods (UPFs) and the energy contribution from UPFs was higher among vegans compared to omnivores [34]. Among vegans, both healthy (h-PDI) and unhealthy (u-PDI) plant-based diet indices were higher compared to omnivores, pesco-vegetarians, and vegetarians [34], providing further evidence of heterogeneity of vegan diets. Consumption of some plant-based foods, such as white potatoes, refined grains, sugar-sweetened beverages, sweets and desserts, and salty snacks was associated with higher risks of major chronic diseases and mortality among non-meat eaters [35,36,37].

New vegans may be especially prone to having unhealthier diet patterns characterized by a higher consumption of UPFs [34], some micronutrient deficiencies, and inadequate nutrient intakes; these are often young people who embrace veganism [38,39,40] as a way of life without sufficient knowledge of healthy eating and the potential risks of prolonged inadequate nutrient intakes. They may also have misconceptions regarding fortified foods (e.g., avoiding use of iodine-fortified salt) or rely on ultra-processed products containing excessive amounts of sodium, added sugar, saturated fats (e.g., savory or sugary snacks, and coconut oil-based products), and additives [34,41,42]. Another source of variation in diet quality might stem from the motivation to become vegan in the first place. At least two subtypes of vegans [43] have been described in vegan subculture: “holistic vegans”, who primarily focus on political and ethical issues related to use of animal products beyond personal diet; and “health vegans”, whose focus is primarily on physical health and longevity. Having evidence-based, easily accessible, and quick-to-use tools for (self-)evaluating vegan diet intakes can help assess diet quality and guide dietary choices in this rapidly growing, potentially nutritionally vulnerable population.

Diet quality is a multidimensional construct developed in nutritional epidemiology to evaluate dietary patterns and their associations with the health outcomes or effectiveness of dietary interventions [44,45]. It includes four dimensions: adequacy, balance, moderation, and variety (of healthy dietary components) [46]. Some of the important features of a high-quality diet are that it provides nutrient adequacy, limits the risk of non-communicable diseases (NCDs), and is environmentally sustainable [47]. Diet quality in a population is typically described in the form of evidence-based dietary guidelines and measured by diet quality metrics, such as diet indices or scores. Diet screeners are short-form diet assessment tools [48] aimed at the rapid assessment of overall diets (e.g., rPDQS [49]) or their components (e.g., a fruit and vegetable screener [50]). They typically consist of indicators that distinguish between low and high intakes of foods or nutrients of interest and rank individuals according to frequency of intakes, but they are not intended to estimate the absolute intakes of nutrients or foods [51]. For an indicator to be useful, it should focus on dietary components that are commonly consumed in a population (e.g., sugar-sweetened beverages/SSBs are commonly consumed in Europe) with some degree of between-person variation in intakes (e.g., some individuals frequently consume SSBs, while others do so more rarely or not at all) [51], and that these dietary components represent either important sources of nutrients in question (e.g., SSBs are an important source of added sugar among Europeans) or that are associated with NCD risk (e.g., SSBs are associated with a higher risk of adverse cardiometabolic outcomes).

The VEGANScreener project [52] is a JPI HDHL ERA-NET-funded initiative involving five scientific partners from European countries (Austria, Belgium, Czech Republic, Spain, and Germany), with additional collaborators from the U.S. and Switzerland. Its primary objective is to develop and evaluate a diet quality screener for European vegans. This tool aims to be straightforward for both vegans and non-dietitian/non-nutritionist healthcare providers to use and interpret. Its potential applications include estimating the overall diet quality of vegans, identifying potential areas for dietary enhancement, assisting vegans and their health advisors in setting dietary goals, and monitoring vegan diet quality at both individual- and population-levels over time. This methodological manuscript describes the process for developing the VEGANScreener, a brief tool for assessing diet quality among European vegans.

## 2. Methods

### 2.1. Approach to Diet Quality-Screener Development

**Figure 1:**
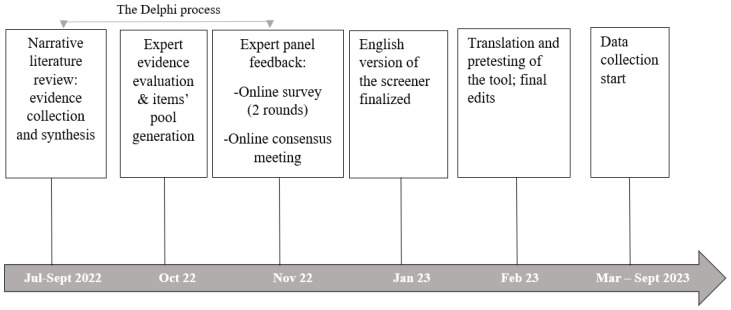
The VEGANScreener development process flow diagram.

To develop the VEGANScreener, we combined approaches from diet quality metrics development with measurement scale methods in behavioral and health sciences. Our final approach, in brief, consisted of three distinct stages: (a) diet and health narrative literature search and synthesis, (b) evaluation of evidence by a group of international experts, and (c) translation of evidence into a measurement tool within pre-identified domains of the construct of interest. Diet quality metrics, such as indices and scores, are a priori defined measures developed on the basis of the current knowledge on diet-disease relationships [53]. To measure a complex construct of diet quality, we also relied on the best practices for development of scales as health and social research tools [54] that involve the development of a pool of items within a number of predefined domains by a team of experts [55], and their transformation into measurable indicators. To formalize the expert opinion collection procedure, we adopted a modified Delphi technique [56,57], a formal process for gaining consensus through controlled feedback from a group of experts on a subject. This technique, which was originally developed in the 1950s and is increasingly used in health research, is especially suitable in situations where there is limited evidence on a topic, when experts are geographically dispersed, when there is a need to mitigate the risks associated with groupthink, and where a clear and documented methodology for achieving consensus is required. It is an iterative process traditionally consisting of a large number of feedback loops, while modified Delphi process versions are more efficient, with only two to three rounds of voting to the initially proposed pool of items [58]. In our study, the modified Delphi process consisted of an item-generation phase, two rounds of anonymized expert-feedback collection, and an online “face-to-face” consensus group meeting where remaining issues were discussed and resolved. For practical reasons (experts operating in different time zones), participation at the final meeting was not mandatory. Figure 1 presents a visual overview of the screener development process.

### 2.2. Data Collection and Analysis

**Figure 2:**
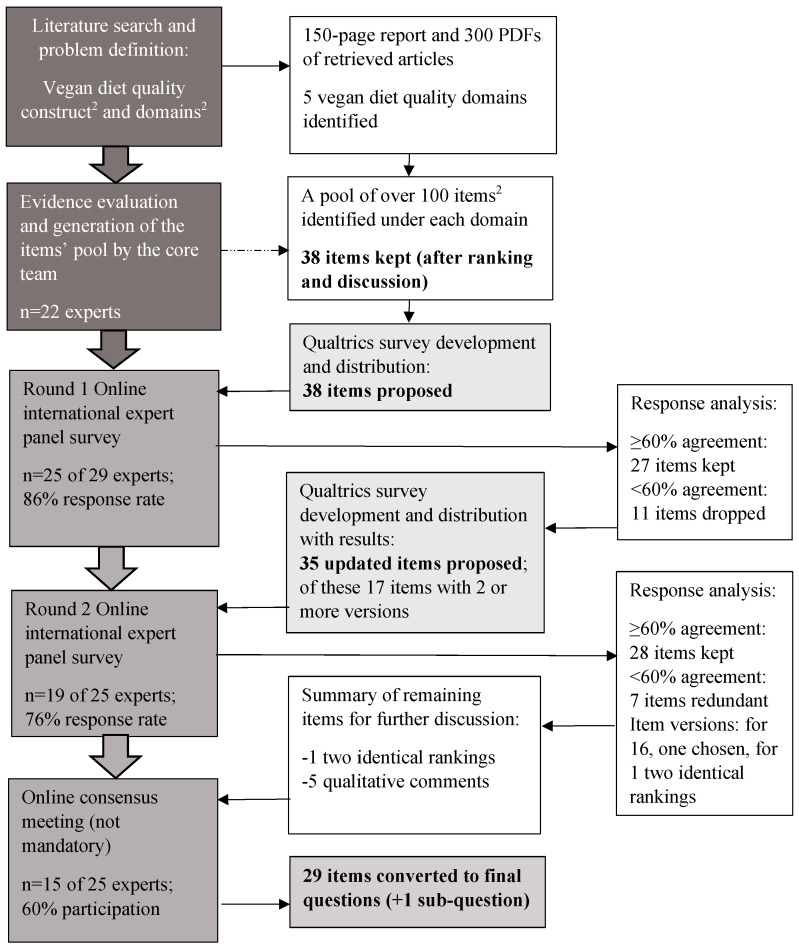
Summary flow of the modified Delphi method process ^1^. (^1^ Adapted from Figure 5 in Taylor et al. [57]. ^2^ Construct, an abstraction representing a phenomenon that is not directly measurable; domain, a subcategory or dimension of a construct; item, a measurable indicator formulated as a question or a statement).

Between April and September 2022, our project team collected and synthesized evidence on associations of plant-based and vegan diets with nutrient intake adequacy, non-communicable diseases and planetary health, diversity of vegan diet patterns, nutrient composition of novel vegan products, and metabolomic profile of vegans. We also collected data on currently existing plant-based and vegan diet guidelines, metrics, and diet assessment tools and identified literature gaps. Finally, we reviewed the existing approaches to diet screener development. Our findings were summarized in a comprehensive report with a database of over 300 retrieved articles and analyses of our existing data on vegans from Germany and the Czech Republic. The Delphi process (Figure 2) consisted of two stages: during stage 1, in October 2022, a “core team” of 22 project partners and collaborators from seven countries (Austria, Belgium, Czech Republic, Germany, Spain, Switzerland, and the U.S.), consisting of nutrition scientists, clinicians, and epidemiologists with expertise in nutritional epidemiology and dietary assessment tools, reviewed the evidence. A number of principles were agreed upon a priori:

- Items should be considered for inclusion based on available evidence on their (a) associations with nutrient adequacy and/or health outcomes, (b) frequency of consumption, and (c) between-person variation in intakes [51];
- The screener should include no more than 30 questions, and these questions should ideally be food group-based. However, given that the nutritional needs of vegans cannot be met without the addition of supplements/fortified foods to their diet, we agreed that questions on the use of supplements or fortified foods could be nutrient-based;
- Food group-intake questions should include a frequency-based answer scheme and some indication of portion size [51];
- Items receiving less than 60% agreement to keep/keep with modifications in the first voting round will be dropped in their original form; any qualitative feedback (i.e., panel comments in free text form) will be carefully reviewed with a possibility for reintroducing the item in a different form depending on presented arguments.
- Items receiving less than 60% agreement to keep (i.e., 40% or more agreement on redundancy) in the second round will be deemed redundant and will be dropped.
- Qualitative feedback in both rounds will be carefully evaluated and incorporated whenever possible.
- Any remaining issues will be discussed at the consensus meeting.
- The screener should include a balance of healthy and unhealthy food group-based questions and should also include a necessary minimum of nutrient-based questions relevant for vegans.

Based on the narrative literature review, the team coordinated by the lead author identified five domains of diet quality: (1) foods/food groups associated with a lower NCD risk, (2) foods/food groups associated with a higher NCD risk, (3) foods/food groups associated with nutrient (in)adequacy in vegans, (4) supplements/fortified foods supplying nutrients otherwise deficient in vegan diets, and (5) other food-related behaviors that may influence vegan diet quality. Then, under each of these domains, core team members proposed individual items. Finally, core team members ranked the proposed items by importance by assigning 0, 1, or 2 points to each of them. The summed-up values were then discussed in a plenary meeting, and the final items’ pool for further review in stage 2 was adopted. In stage 2, 29 experts in nutrition, epidemiology, and preventive medicine, with backgrounds in plant-based diets or diet assessment (including 10 core team members), were invited via email to participate in a month-long evaluative process. This process was segmented into two distinct rounds of feedback utilizing a Qualtrics-based survey [59]. This method ensured the mutual anonymity of the responses, adhering to the Delphi technique’s principles [56,57,58]. Experts were also invited to participate in the wrap-up online meeting where any eventual remaining issues would be discussed and resolved.

During the initial round, the experts were provided with a link to the Qualtrics survey containing the item pool. Simultaneously, they were given the summary of evidence report and the full texts of collected articles that were reviewed in stage 1. The experts were asked to review the proposed pool of items and opt for one of three designated responses: “include”, “exclude”, or “keep with modifications”. In the case of the third option, experts were asked to suggest specific modifications. In addition, experts were given an opportunity to include qualitative feedback about each item. For any item to transition to the subsequent round, it necessitated a consensus of at least 60%. Any qualitative feedback, including comments about the dropped items, was carefully reviewed to ensure all feedback was taken into account. A separate team, not involved in the voting process, appraised these responses, resulting in refined items or, in instances of divergent feedback, the genesis of multiple versions of an item.

In the second round, the experts were provided with a new Qualtrics survey link and summaries of the responses accumulated during the first round. This also included an updated pool of items, with certain items presented in multiple iterations. The experts were then asked to vote on the necessity or redundancy of each item. For those items existing in multiple versions, a ranking criterion was implemented to gauge preference. The voting process also requested experts to consider the comprehensive composition of the screener, ensuring the balanced representation of all relevant food groups and nutrients. Items or their specific versions that garnered an agreement threshold of 60% were marked for retention. Residual concerns, whether they related to ranking ties or feedback from the second round that warranted additional consideration, were taken forward for discussion at the online meeting.

The Delphi process was quasi-anonymous [57]; while panelists knew the names of the participating panel members, there was no interaction among the panel members during the first two rounds, and all panelist responses were anonymized.

### 2.3. Translation and Pretesting

Once finalized, the screener version in English was translated to Czech, Dutch, German, and Spanish and pretested. Our translation process followed the guidelines from the International Society for Pharmacoeconomics and Outcomes Research (ISPOR) [60]. It included translation to local languages by native speakers in target languages, back-translation by English native speakers, and a comparison of the two versions (the original and the back-translated version in English) to detect any semantic shifts. Any spotted shifts were corrected by a team of reviewers and submitted for another round of translations, back-translations, and revisions until the local version corresponded to the original in English. Local versions of the screener were then pretested for clarity and ease of completion in a small convenience sample of vegans, vegetarians, and omnivores (at minimum, ten individuals for each language version). Qualitative feedback was recorded and shared with the core team for review and discussion. Any agreed changes were made to the master version in English, which was then taken for the next round of translations. The procedure was repeated until all local versions were understandable and corresponded with the master version in English. When completed, local language versions were entered into REDCap [61], a research platform used for building and managing online surveys and databases, suitable for multi-country projects.

## 3. Results

### 3.1. Voting Rounds and Feedback

The core team reviewed a pool of over 100 items extracted during the literature search and decided to keep 38 for the expert voting. After consideration of the 38 items by the panel of 25 experts participating in round 1 (25 out of 29, 86% response rate), 27 items received agreement of 60% or more and were kept for the next round, while 11 were dropped. The project team then reviewed and summarized qualitative feedback, and it was used to enhance the “kept items” and to potentially restructure the dropped items. As a result, 35 updated items (of which there were 17 with two or more versions) were proposed for round 2 of expert voting. In total, 19 experts participated in round 2 (19 out of 25, response rate 76%); 28 items received ≥60% agreement and were kept, while 1 was taken forward (due to identical agreement for both versions) to the online consensus meeting for discussion. All qualitative feedback from this round was carefully considered by the project team and incorporated for further consideration. Finally, 15 experts (15 out of 25, 60% participation rate) took part in the online consensus meeting, where remaining dilemmas were resolved, and the items were converted to 29 questions and one sub-question, creating the final screener draft.

### 3.2. Examples of Qualitative Feedback Incorporation

Special attention was given to qualitative feedback provided by experts and their incorporation for consideration by the panel in the next round. Between the first and the second round, a number of items were reformulated, and some merging of items, as well as splitting of items, took place as well (Table 1). For example, the initial item “intake of vegetables (any, except potato)” was deemed insufficiently specific by experts and received low agreement and a number of modification suggestions; “green vegetables” with country-specific lists was deemed overly complex, and experts called for a single list; and the “fruit and vegetable smoothies” item was dropped with a suggestion from some experts to add vegetable smoothies to the general vegetables item. As a result, in round 2, three items (two with multiple versions) were proposed. The final result of this process was three separate questions on “green vegetables”, “other vegetables”, and “dark orange and red fruits and vegetables”. Table 1 includes several examples of the “evolution” of the initially proposed items into screener questions.

### 3.3. Examples of Changes Made during Translation and Pretesting

Between 10 and 72 respondents provided feedback on each language version of the questionnaire. Overall, participants found the screener straightforward and easy to complete. In some cases, respondents were unsure whether certain foods should be included in the response. In such cases, their comments were recorded and discussed by the core team, who gave potential solutions. For example, some respondents were unsure whether chickpea or red lentil flour products (e.g., red lentil pasta) should be reported under the question on legumes. As a result, this question was slightly reformulated to include products made of legume flour. Another example was editing the list of commonly consumed calcium-rich, oxalate-low vegetables in countries; while in some countries, bok choy was commonly consumed, it was not the case across all countries. At the same time respondents suggested adding other locally consumed green leafy vegetables such as endive or borage. Finally, respondents noted that the sub-question on the salt content of vegan meat alternatives could be difficult to answer with only “yes” and “no” options, as some respondents might not know whether they were choosing it or not. Given that our team’s intention was to capture those who take care to choose products not overly high in sodium, we added a response “do not know” to the answer scheme, assuming that those who do not intentionally choose low-sodium products from the meat alternatives category are most likely consuming sodium-rich ones [62,63,64].

### 3.4. Final Screener Draft for Evaluation and Testing

The final screener draft (Table 2) consisted of 29 questions and one sub-question; of these, 17 questions focus on intake of food groups and nutrients whose intake should be encouraged (e.g., wholegrain bread, bun, roll, crisp, or crackers) and 12 (+one sub-question) on intake of food groups that should be limited (e.g., white bread, bun or roll); 24 were food-based (e.g., sugar-sweetened beverages), and 5 were nutrient/supplement-based (e.g., vitamin B12 supplement). The rationale for including each screener question in its final form is presented in Table 3. One question could cover one or more reasons for inclusion; for example, nuts and seeds are good sources of many micronutrients important for vegans, as well as of amino-acids and PUFAs. Twenty-five food group intake questions had a frequency-based format including nine possible answer options (from “never” to “3 or more times a day”) and an example of one serving (e.g., one cup), four nutrient-based were binary (yes/no), and one sub-question had a “yes/no/don’t know” answer format.

### 3.5. Challenges in Development of the Screener

Ensuring a highly rigorous process of development of the screener, following an a priori defined procedure and basing any decisions on available scientific evidence was our team’s priority at each stage of the process. We identified and followed best-practice steps for tool development described in the scholarly literature, conducted a comprehensive narrative literature review on aspects of vegan diets and health, and invited participation of a diverse group of international experts. During this process, however, we encountered several important challenges. The vegan food market in Europe is growing rapidly, resulting in multiple data gaps regarding the nutrient composition and health effects of these foods. As a result, we found it difficult to classify some of these novel products under traditional food groups; categorize them as “healthy” and “less healthy” food groups; and to formulate them as clear, simple, and understandable screener questions. For example, vegan cheese alternative can be based on legumes (considered “healthy”) or on coconut and shea butter fat (considered “less healthy”). Therefore, when we aimed to measure how often respondents consumed “less healthy” vegan cheese, we formulated it as follows: “cheese alternatives containing coconut oil, such as…”, assuming that respondents would know what type of cheese alternatives they use, which might not always be the case. Another similar problem was related to fortified milk alternatives, as they can be any combination of calcium-fortified/unfortified and “sugar-added”/“no added sugar”. As we aimed to capture only intakes of “healthy” calcium-fortified milk with no added sugar, we formulated the question by emphasizing calcium-fortification and combining milk with other dairy alternatives such as calcium-fortified yoghurt and calcium-set tofu, leaving out mention of sugar, as some experts felt that that would be overly complicated and confusing for participants. The lack of data on long-term effects of some foods was another challenge. As already mentioned, some vegan cheese or vegan butter products are coconut oil- and/or shea butter-based and contain high amounts of saturated fats, making them potentially unhealthy [65]. Some vegan meat alternatives and snacks may also contain excessive amounts of sodium [62,63,64]. Finally, while many of these novel products are classified as ultra-processed foods [34,66], some of which are associated with adverse health outcomes in large studies [67,68], others are not [69].

## 4. Conclusions

After extensive collaboration and evaluation by experts, we successfully developed the VEGANScreener, a diet quality screener tailored for vegans in Europe. The next steps involve validating the tool against reference diet assessment methods and nutritional biomarkers across different European population groups and settings, with special paid attention to evaluating any variations by gender, age, ethnicity, or motivation for becoming vegan. Its performance and acceptability will also be evaluated among both vegans and healthcare professionals. If deemed valid and well-received, the VEGANScreener could become a straightforward tool for the self-evaluation, monitoring, and enhancement of dietary quality among European vegan individuals and groups. It also holds potential for developing a similar tool for other geographical settings through the application of the process described in this manuscript.

## Figures and Tables

**Table 1:** Transformation of initial items into final screener questions: selected examples.

Proposed Items in Round 1	Round 1 Voting Whether Items Should Stay In	Proposed Items in Round 2	Round 2 Voting	Final Screener Questions
A01. How often do you consume vegetables (excluding potatoes and legumes) A02. Consumption of the following green vegetables: → list of calcium-rich commonly consumed vegetables in each country A04. Consumption of fruit and vegetable smoothies (include only freshly made ones)	<60% agreement on all items regarding their retention Qualitative feedback for modifications	A02.1. Consumption of the following green vegetables, fresh or cooked: ○ Broccoli ○ Kale ○ Bok choy ○ Celery sticks ○ Arugula ○ Green cabbage ○ Chinese cabbage A02.2. The following green vegetables, fresh, cooked, or in smoothie: ○ Broccoli ○ Kale ○ Bok choy ○ Celery sticks ○ Arugula ○ Green cabbage ○ Chinese cabbage A07. The following dark orange and red fruits and vegetables: ○ Carrot ○ Apricot ○ Pumpkin ○ Sweet potato ○ Butternut squash ○ Winter squash ○ Cantaloupe ○ Red bell pepper ○ Mango A01.1. Vegetables, any (e.g., fresh, frozen, canned, cooked, fried, etc.) (excluding white potatoes and legumes) A01.2. Other vegetables, such as tomato, cucumber, onion, zucchini, or eggplant—fresh, frozen, canned, cooked, fried (do not include vegetables listed in previous question, white potatoes, or legumes)	A02.1: 90% agreement to keep A07: 85% agreement to keep A01.2: 84% agreement to keep Qualitative feedback for further improvement	Q1. The following green vegetables, fresh or cooked; whole, cut, or blended: ○ Broccoli ○ Kale ○ Bok choy ○ Arugula ○ Chinese cabbage ○ Brussel sprout ○ Green cabbage ○ Savoy cabbage ○ Endive ○ Artichoke Q2. The following dark orange and red fruits and vegetables, fresh or cooked; whole, cut or blended: ○ Carrot ○ Apricot ○ Mango ○ Cantaloupe ○ Orange sweet potato ○ Hokkaido pumpkin ○ Butternut squash ○ Dark orange pumpkin ○ Red bell pepper ○ Red grapefruit ○ Kaki ○ Chanterelle mushroom ○ Milkcap mushroom Q3. Other vegetables, such as tomato, cucumber, onion, zucchini, or eggplant—fresh, frozen, canned, cooked, fried (do not include potatoes, legumes or vegetables listed in previous questions)
A24. Sugar-sweetened beverages such as soft drinks, lemonades or sports drinks	76% agreement Suggestions for further improvement	A24.1. Sugar-sweetened beverages such as soft/fizzy drinks, lemonades, sweetened iced tea, flavored plant milk, energy drinks, ginger beer, or sports drinks A24.2. Sugar-sweetened beverages such as soft/fizzy drinks, lemonades, sweetened ice teas, flavored milk alternative, non-100% juice drinks/fruit nectars, energy drinks, ginger beer, or sports drinks A24.3. Sugar-sweetened beverages such as soft/fizzy drinks, lemonades, sweetened ice teas, flavored plant milk/yogurt drink, non 100% juice drinks/nectars, energy drinks, ginger beer, or sports drinks	A24.1: 88% agreement to keep Qualitative feedback for further improvement	Q23. Sugar-sweetened beverages such as soft/fizzy drinks, lemonades, sweetened iced tea, flavored plant-based milk, energy drinks, ginger beer, or sports drinks
A16. Beans, lentils, chickpeas, soybeans, or peas (excluding green peas and green beans)	76% agreement Suggestions for further improvement	A16.1. Beans, string beans, lentils, chickpeas, or peas (excluding green peas and green beans), in stews and salads, excluding their products, such as tofu, tempeh, or hummus A16.2. Beans, string beans, lentils, chickpeas or peas in stews and salads, excluding their products, such as tofu, tempeh, or hummus	A16.1: 95% agreement to keep Qualitative feedback for further improvement	Q16. Beans, soybeans lentils, chickpeas, or peas (excluding green peas and green beans) (do NOT include here their products, such as tofu, tempeh, or hummus)
A20. Consumption of cheese alternatives such as vegan sliced or grated cheese, feta, mozzarella, or cream cheese	83% agreement Suggestions for further improvement	A20.1. Cheese alternative such as sliced or grated vegan cheese (e.g., vegan feta, mozzarella, or cream cheese) A20.2. Cheese alternative, such as vegan sliced or grated cheese (e.g., feta, mozzarella, or cream cheese). Do not include here nut/seed-based vegan cheeses.	A20.1: 89% agreement to keep Qualitative feedback for further improvement	Q20. Cheese alternatives containing coconut oil, such as sliced, solid, or grated vegan cheese (e.g., vegan feta, mozzarella, or cream cheese)
A32. Do you consume seaweed and/or use iodized salt in food preparation and/or an iodine supplement (for Czech Republic, iodine-rich mineral water)	Accepted (over 80% agreement) Suggestions for further improvement	A32.1. Do you use iodized salt in food preparation, use an iodine supplement or consume seaweed to supplement for iodine intake (for Czech Republic also, iodine-rich mineral water) A32.2. Do you consume seaweed to supplement for iodine intake and/or use iodized salt in food preparation and/or an iodine supplement (for Czech Republic, also iodine-rich mineral water) A32.3 Do you supplement iodine (e.g., seaweed, iodized salt, iodine supplement, and Cz/iodine-rich water)	A32.1: 82% agreement to keep Qualitative feedback for further improvement	Q27. Do you regularly use iodized salt in food preparation, use an iodine supplement (either individually or as a part of multimineral supplement) or consume seaweed to supplement for iodine intake (for Czech Republic, also iodine-rich mineral water)

**Table 2:** VEGANScreener—final English version.

Over the Past Month, How Often Did You, on Average, Consume at Least One Serving of Foods or Beverages from the Following Food Groups:				Never	Rarely/ 1×/Month	2–3×/ Month	1×/ Week	2–3×/ Week	4–6×/ Week	1×/ Day	2×/ day	≥3 Times /Day	1 Serving Example	
Q1	The following * green vegetables, fresh or cooked; whole, cut, or blended:												1 handful cooked/ 2 handfuls fresh vegetables	
	Broccoli Kale Bok choy Arugula	Chinese cabbage Green cabbage Savoy cabbage	Brussel sprout Endive Artichoke											
Q2	The following * dark orange and red fruits and vegetables, fresh or cooked; whole, cut, or blended:												2 handfuls fresh/1 handful cooked, 1 medium piece	
	Carrot Apricot Mango Cantaloupe Orange sweet potato	Hokkaido pumpkin Butternut squash Dark orange pumpkin	Red bell pepper Red grapefruit Kaki Chanterelle mushroom Milkcap mushroom											
Q3	Other vegetables, such as tomato, cucumber, onion, zucchini, or eggplant—fresh, frozen, canned, cooked, fried (do not include potatoes, legumes, or vegetables listed in previous questions)												1 medium tomato, 1 handful cooked/2 handfuls fresh vegetables	
Q4	Other fruits, such as apples, berries, melons, or oranges—whole or cut (do NOT include fruit juices and smoothies)												1 medium apple/orange, 1–2 slices melon, 1 handful berries	
Q5	White/yellow potatoes												2-3 medium potatoes	
Q6	White bread, white bun, or white roll												2 slices of bread, 1 bun	
Q7	White rice, pasta/noodles, instant couscous, instant polenta, or instant breakfast cereals (e.g., crisps, flakes, and crunch)												1 cup of cooked rice, pasta, couscous, polenta or instant breakfast cereals	
Q8	Wholegrain bread, bun, or roll, wholegrain crackers or crispbread												2 slices of bread, 1 roll, 2–3 crackers/crispbreads	
Q9	Other whole grains (e.g., brown rice; brown pasta; grain kernels such as spelt, wheat, oats or barley, porridge, unsweetened wholegrain muesli, wholegrain couscous, wholegrain bulgur, quinoa, buckwheat, or amaranth)												1 cup cooked rice, pasta, porridge, kernels, couscous, bulgur, quinoa, buckwheat, amaranth, or muesli	
Q10	Nuts and seeds, such as walnuts, almonds, hazelnuts, pumpkin seeds, sunflower seeds, or flaxseeds												1 handful of nuts, 1 tablespoon of seeds	
Q11	Nut and seed butters, such as peanut butter or tahini												1 tablespoon	
Q12	Vegan butter or coconut oil												1 tablespoon	
Q13	Plant-based oils such as olive, soybean, flaxseed, or rapeseed oil (do NOT include here palm or coconut oil), avocado or olives												1 tablespoon oil, 1/2 avocado, 5–10 olives	
Q14	EPA/DHA (omega 3)—fortified oils, EPA/DHA (omega 3), supplements or microalgae oil?												1 tablespoon oil, 1 dose as per packaging instructions.	
Q15	Traditional plant protein sources and derivates like tofu, seitan, natto, tempeh, falafel, hummus, 100% red lentil or chickpea pasta, or soy cubes/granules												½ small block tofu, seitan, tempeh, 4 falafels;1 cup cooked pasta, 1 small bowl soy granules/cubes, 2–3 tablespoons hummus	
Q16	Beans, soybeans, lentils, chickpeas, or peas (excluding green peas and green beans) (do NOT include here their products, such as tofu, tempeh, or hummus)												1/2 cup cooked legumes	
Q17	Packaged meat/fish alternatives such as vegan salami, cold cuts, sausages, burger patties, or fish fingers (excluding homemade recipes from raw sources) If 17 = “never”, go to Q18												1 palm-sized piece, 1 sausage, 3–4 slices of salami etc.	
Q17 a	When you buy these products, do you usually choose products low in salt?			Yes	No	Don’t know								
Q18	Calcium-fortified plant-based milks, yogurts (e.g., almond, soy, and oat) or calcium-set tofu												1 glass of milk, 1 cup yogurt, ½ small block of tofu	
Q19	Cheese alternatives containing coconut oil, such as sliced, solid, or grated vegan cheese (e.g., vegan feta, mozzarella, or cream cheese)												1 slice of cheese, 1 tablespoon cream cheese or grated cheese	
Q20	Savory snacks, such as crisps/chips or salted crackers												1 handful	
Q21	Ready-to-eat meals, such as frozen pizza, croquettes, fried foods, spring rolls, dumplings, instant pasta, or instant soup												1 serving according to the package	
Q22	Vegan sweets and desserts, such as candy, “milk” chocolate, cake, ice cream, or pudding												1 piece of cake, 4 cookies, 1 handful of candy, 1 rip of chocolate, 1 scoop ice cream, 1 bowl of pudding	
Q23	Sugar-sweetened beverages, such as soft/fizzy drinks, lemonades, sweetened iced tea, flavored plant-based milk, energy drinks, ginger beer, or sports drinks												1 glass	
Q24	Artificially sweetened beverages, such as diet/zero sugar soft/fizzy drinks, lemonades, energy drinks, “light” beverages, or sports drinks												1 glass	
Q25	Alcoholic beverages such as beer, wine, cocktails, or spirits												1 can, 1 glass, 1 jigger/shot	
	Do you regularly use:												Yes	No
Q26	Supplement for vitamin B12 (either individually or as part of a multivitamin supplement) (e.g., pills, drops, injections, and fortified toothpaste)?													
Q27	Iodized salt in food preparation, use an iodine supplement (either individually or as part of a multimineral supplement) or consume seaweed to supplement for iodine intake (for Czech Republic, also iodine-rich mineral water)													
Q28	Vitamin D supplement (either individually or as part of a multivitamin supplement) during autumn and winter months?													
Q29	Selenium supplement (either individually or as part of a multimineral supplement) or regularly consume Brazil nuts?													

* These lists may be edited by adding country-specific fruits/vegetables if they are commonly consumed in a country AND if they contain >40 mg Ca/100 g AND have a low oxalate content (due to inhibited Ca absorption).

**Table 3:** Rationale for inclusion of each question in the VEGANScreener.

VEGANScreener Question (Abbreviated Title) *	Rationale for Inclusion and Format
Green vegetables	Critical nutrient (calcium) List of vegetables satisfying the following three requirements: Calcium content > 40 mg/100 g Commonly consumed in country Low oxalate content
Dark orange and red fruits and vegetables	Beta-carotene intake List of fruits and vegetables satisfying the following three requirements: Beta-carotene content > 130 mcg/100 g Commonly consumed in country
Other vegetables	Dietary fiber and micronutrient intake, diverse vegetable intake
Other fruits	Dietary fiber and micronutrient intake, diverse fruit intake
White/yellow potatoes	High glycemic index, pro-inflammatory
White bread, white bun, or white roll	High glycemic index, pro-inflammatory
White rice, pasta/noodles, instant couscous, etc.	High glycemic index, pro-inflammatory
Wholegrain bread, bun or roll, etc.	Dietary fiber and micronutrient intake
Other whole grains	Dietary fiber and micronutrient intake
Nuts and seeds	Polyunsaturated fat, plant protein intake and, micronutrient intake
Nut and seed butters	Polyunsaturated fat, plant protein intake, and micronutrient intake
Vegan butter or coconut oil	Saturated fat content, pro-inflammatory
Plant-based oils	Long-chain omega 3 fatty acids intake
EPA/DHA-fortified oils or supplements	EPA/DHA intake
Tofu, seitan, natto, tempeh, etc.	Plant protein intake
Beans, soybeans, lentils, chickpeas, or peas	Dietary fiber and plant protein intake
Packaged meat/fish alternatives	Saturated fat and sodium content
Salt content (sub-question, refers to previous question)	Potentially high sodium content
Calcium-fortified plant-based milks, etc.	Calcium and plant protein content
Cheese alternatives containing coconut oil	Saturated fat content
Savory snacks	Saturated fat and sodium content
Ready-to-eat meals	Saturated fat added sugar and/or sodium content
Sweets and desserts	Saturated fat and added sugar content
Sugar-sweetened beverages	Added sugar content
Artificially sweetened beverages	Associations with adverse CVD outcomes and possibly cancer
Alcoholic beverages	Inhibiting nutrient absorption
Use of supplement for vitamin B12	Critical nutrient for vegans
Use of iodized salt, supplement, or seaweed	Critical nutrient for vegans
Use of vitamin D supplement	Critical nutrient for vegans
Use of selenium supplement	Critical nutrient for vegans

* Refer to Table 2 for a full list of questions.

## Data Availability

All data generated or analyzed during this study are included in this published article.
